# The role of relative age effects in generic motor skill diagnostics using the German motor test (6–18): diagnostic outcomes and recruitment of 9–10-year-old children to sports schools in Germany

**DOI:** 10.3389/fpsyg.2026.1814502

**Published:** 2026-05-08

**Authors:** Isabel Stolz, Tabea Bokeloh, Chiara Feldhaus, Klaus Bös

**Affiliations:** 1 Institute of Movement and Neurosciences, German Sport University Cologne, Cologne, Germany; 2 Research Center for Physical Education and Sport for Children and Adolescents, Karlsruhe Institute of Technology, Karlsruhe, Germany

**Keywords:** athlete development, diagnostics, elite sports, generic motor skill diagnostic, performance development, youth sports

## Abstract

**Introduction:**

Generic motor skill diagnostics are widely applied in early talent identification alongside anthropometric and sport-specific performance measures. However, talent selection processes in youth sports are frequently influenced by relative age effects (RAEs), which are closely linked to diagnostic procedures and recruitment practices.

**Methods:**

This retrospective analysis examined associations between calendar age and motor performance in a large cohort of German fourth-grade schoolchildren (*N* = 19,179; aged 9–10 years). All participants completed the German Motor Test 6–18 (GMT 6–18) between 2008 and 2022 as part of the diagnostic process for recommendations to North Rhine-Westphalia (NRW) Sports Schools, a combined school and competitive sport system. Motor performance differences were analyzed using analyses of variance, reporting *F-*values, *p-*values, and effect sizes (η^2^). Furthermore, generalized linear models (GENLIN) were applied to examine associations while accounting for covariates and to support the robustness of the findings. Recommendation and admission rates were assessed using Pearson's chi-square tests, Cramer's *V*, corresponding *p-*values and a logistic regression analysis.

**Results:**

The results indicate that the magnitude of RAEs in this sport-unspecific, generic motor skill assessment was comparatively low within the 9–10-year-old age group. While statistically significant differences between age groups were observed, effect sizes were consistently small, suggesting limited practical relevance. Overall, the results indicate that raw values should be considered in addition to effect sizes for an appropriate interpretation of age-related differences.

**Discussion/Conclusion:**

The gained findings show few meaningful differences in motor performance between age groups, but a practically relevant RAE can be negated. Conclusively, suggestions are given as to how the RAE could be countered in diagnostics to further reduce potential RAEs in talent diagnostics, including refined analytical approaches and mathematical adjustments.

## 1 Introduction

Generic motor skills diagnostics are applied in early and middle childhood to identify talent. Test items from the German Motor Test (GMT 6–18) are implemented across various diagnostic and talent identification contexts alongside sport-specific test parameters to identify children with above-average motor potential and to support the optimal development of athletic talent (Hohmann and Siener, 2021; Roth et al., 2019; State Government of North Rhine-Westphalia, Germany, 2026; Siener, 2021). In this regard, generic motor skill assessments are not only considered merely as preliminary screening tools but could also serve as indicators of early sport-specific performance dispositions that may manifest in later athletic success (Hohmann and Siener, 2021). Within the *generality–specificity dilemma* of talent identification, current evidence supports the notion that generic assessments are particularly informative at developmental stages in which sport-specific skills are not yet fully differentiated (Pion et al., 2020; Pion, 2015). By capturing fundamental neuromotor and physical capacities that underpin later sport-specific performance, a generality-first diagnostic approach could provide developmentally appropriate information and may improve long-term talent prediction while reducing premature specialization and selection bias. From a theoretical standpoint, this perspective aligns with the conceptual framework of the *Mountain of Motor Development* (Clark and Humphrey, 2002). Accordingly, motor development is understood as a non-linear process emerging from the interaction of individual characteristics, environmental conditions, and task demands (Clark and Humphrey, 2002). Although developmental pathways differ between individuals, motor development follows a cumulative progression in which earlier movement experiences provide the foundation for later, more specialized performance (Clark and Humphrey, 2002). Importantly, advancement within this process appears to be more closely associated with developmental maturity and prior movement experience rather than chronological age. Accordingly, generic motor assessments in childhood seem to offer a developmentally appropriate approach to uncover individual motor capacities and may be informative for identifying athletic potential. A central criticism directed at sport-specific assessment inventories concerns the substantial heterogeneity in study design parameters, resulting in an ambiguous body of evidence regarding the prognostic validity of physiological tests targeting both generic motor abilities and sport-specific technical skills (Hohmann and Siener, 2021).

Nevertheless, generic motor skills diagnostic requires systematic consideration of potential influencing factors and sources of bias to ensure equitable access to sport-related development and talent promotion programs for children. In this context, relative age effects (RAEs) represent a relevant factor to examine, since they describe the phenomenon that children born at the early part of a year tend to have an advantage to be admitted to competitive sport structures due to the relative and absolute age and development advantage over same-aged peers (Ferrauti, 2020; Wattie et al., 2015). Regarding generic motor skills diagnostics, the potential that older children within an age cohort may achieve superior test outcomes due to age-related advantages in motor development rather than underlying differences in athletic talent merits consideration. The occurrence of RAEs has increasingly shifted into a scientific focus in recent years, measured by the number of Medline-listed publications on this topic, which has doubled in the last 5 years (NCBI Pubmed Database, n.d.). The effect is evident in competitions or selection processes in which the date of birth plays a role, such as in elite youth sports teams or to be recruited to sports academies (Gil et al., 2021; Votteler and Höner, 2017). Evidence for the presence of RAEs has been provided for competitive team sports such as soccer, handball, basketball, rugby and hockey (Tascioglu et al., 2023; Bozděch et al., 2023; de La Rubia et al., 2020b; Kelly et al., 2021) and for strength- and speed-oriented individual sports, such as athletics, judo and skiing (Brustio et al., 2023; Fukuda, 2015; De Larochelambert et al., 2022). Additionally, a reversal in the senior transition was observed in some sports (Bozděch et al., 2023; Kelly et al., 2021; Brustio et al., 2023). However, the mechanisms underlying RAEs are complex and context dependent (Wattie et al., 2015). It requires a differentiated perspective that accounts for diagnostic, developmental and psychosocial plus structural influences (Wattie et al., 2015). Depending on gender and age, personal developmental differences vary, such as biological maturity and training age (Müller et al., 2018; Sedeaud et al., 2025). Furthermore, the observation of short-term or long-term performance and success shows different degrees of influence by RAEs, as well as individual or collective performance in team sports or playing positions (Wattie et al., 2015; Tascioglu et al., 2023; de La Rubia et al., 2020a). Leyhr et al. investigated the extent of the relationship between 16,138 soccer players' chronological and relative age in different age groups (U12 to U15) plus objective and subjective performance assessments (Leyhr et al., 2021). They found an overrepresentation of early-born players in all age groups and correlations between the chronological age of players and anthropometric parameters, speed abilities and technical skills (Leyhr et al., 2021). However, when evaluating each age group separately, small effects were found correlating relative age with anthropometry, speed abilities and technical skills (Leyhr et al., 2021). In this regard, Gil et al. found that chronological age was the most important variable in the agility test and the overall score in youth soccer player diagnostics (Gil et al., 2014). The results comparing different age cohorts show that RAEs become increasingly relevant from puberty to adult age groups (Bozděch et al., 2023). Studies indicate that RAE bias in child samples (U9, 9–10 years old or 9–11 years old) may be less practically relevant and/or influenced by maturity status, which is especially the case later in puberty age groups (Dutil et al., 2018; Müller et al., 2018). Controversy, the current results of the EUROFIT study suggest that older children within the same age cohort (9–11 years old) tend to show greater explosive strength, speed, and power with overall heterogeneous test results, reflecting persistent variability in the existing evidence (Kryeziu et al., 2025). Researchers argue that the validity and reliability of measures need to be considered in a more discriminating way to predict individual future performance in youth sports and talent identification programs (TID) (Bergkamp et al., 2019; Johnston et al., 2018). A test battery with several strength-related test items led to a superior performance of the relatively older ones, even in elementary school years (Sandercock et al., 2013; Drenowatz et al., 2021).

A methodological issue that arises when using comprehensive test batteries is the use of standardized *Z*-scores to compare physical performance. Rounded down values of whole-year age could lead to flattening out natural differences in physical development, resulting in RAEs appearing less pronounced or differently pronounced in standardized data than actually occurs in real performance measurements (Sandercock et al., 2013). In this regard, the specification of strength and power indices per unit of body mass could enable a more precise comparison between boys and girls in the age range from 10 to 16 years old (Sandercock et al., 2013). In this regard, it seems to be beneficial to also take a closer look at raw values of test items when using test batteries to determine the actual performance of children born in the same year.

The present study examined the possible correlation between RAEs and Generic Motor Skill diagnostics using the GMT 6-18 in the combined system of competitive sport structures and the German school system, focusing on both standardized Z scores and raw values. In Germany, a united system exists in the state North Rhine-Westphalia (NRW), which focuses on youth development in so-called “NRW sports schools” (State Government of North Rhine-Westphalia, Germany, 2026). NRW Sports Schools aim to offer talented young athletes a special education that combines both sporting development and school education. The aim is to promote talented athletes at the highest level by combining their sporting skills with a qualified school education to pursue a sporting career while at the same time achieving a school-leaving qualification. Pupils have 2 h of additional athletics training per week supplemental to their regular physical education lessons to develop their athletic talent in the best conditions. Additionally, a career as a competitive athlete is greatly academically supported in these combined structures (State Government of North Rhine-Westphalia, Germany, 2026).

From a research perspective, an interconnected system that combines school and sport as an integrated support structure is of particular importance for scientific monitoring because talent selection processes have much more complex consequences for children and adolescents in elite sports; therefore, a possible bias would not only lead to a sporting disadvantage but also to a disadvantage at school for affected children. For entry, talented children take part in the GMT 6–18 at the end of elementary school as an entrance test at the age of 9–10 and must achieve a score of 820 points (total Z score) to receive a recommendation for an NRW sports school to enroll (the Z score is based on gender and calendar age). However, the schools can set their own thresholds above the 820 points for actual admission, for example, in the case of a high number of applicants or a higher level of performance. The GMT 6–18 represents a widely established and comprehensive test for the assessment of motor performance of (school) children in Germany, applicable from 6–18 years (Bös, 2017). It comprises 8 exercises on coordination, strength, speed, flexibility and endurance and was developed by Bös et al. with an expert group (Bös, 2017). With the help of this aptitude analysis, children with particularly promising motor performance are to be identified and admitted to sports schools in NRW to optimize the development of their sporting talent (Sporttalents NRW, n.d.). When applied in selecting talent in the interconnected system, GMT is decisive at an early age (9/10 years old). The GMT is introduced in more detail in the methods section.

This retrospective study aims to examine the role of RAEs in generic motor skill diagnostics using the GMT 6-18 and its impact on diagnostic outcomes and recruitment of 9–10-year-old children to sports schools in Germany. The research question was to what extent RAEs were associated with diagnostic outcomes in generic motor skill assessments using the German Motor Test (6–18) and with the recruitment of 9–10-year-old children to sports schools in Germany. Based on the available research data for the age group 9–10, it was hypothesized (1) that there is no meaningful correlation between calendar age and motor performance based on GMP 6–18 in 9–10-year-old children (assumed for the total *Z*-score and the raw values of the individual test items). Furthermore, it was hypothesized (2) that there was no dependency between calendar age and the recommendation for and admission to an NRW sports school.

## 2 Materials and methods

The results presented are based on a retrospective analysis of original data sets. The analysis of data was approved by the Ethics Committee of German Sport University Cologne in accordance with the Declaration of Helsinki, 7th revision.

### 2.1 Participants

A total of 19.179 fourth-graders aged between 9 and 10 years from NRW were included in the analysis: 12,538 males (65.4%) and 6,641 females (34.6%). At the time of the test, 12,167 were 9 years old (63.4%), male = 7,968 (65.5%), female = 4,199 (34.5%) and 7,012 were 10 years old (36.6%), male = 4,570 (65.2%), female = 2,442 (34.8%). Most of the included children are born in *G*4 (9.75–9.99 years old) and *G*5 (10.00–10.24 years old) and therefore are either “older” 9-year-olds or “younger” 10-year-olds, as the assessments are conducted annually between September and March in preparation for entry into secondary school in the following year. The resulting age distribution is shaped by the extended assessment period in combination with the subsequent school enrollment process. Data collection was carried out in the years 2007/2008 to 2021/2022 within the Motor Test 1 project (MT1) of the Research Centre of School Sports and the Physical Education of Children and Young Adults (FoSS) at Karlsruhe Institute of Technology (KIT). The enduring project is carried out on behalf of the state chancellery of North Rhine-Westphalia (NRW), Germany. Children specifically entered GMT 6-18 at the respective NRW sports school and gave written informed consent as part of the application.

### 2.2 Materials and measures

GMT 6-18 (Bös et al., 2021) was applied for the whole sample, which has been validated for children and adolescents aged 6–18 years. The test fulfills the quality criteria of objectivity, reliability and validity and represents an established instrument for the assessment of motor performance in schools in Germany (Bös, 2017). It comprises 8 test items: 20-m *sprint* (SP) represents speed, resp. speed of action, and *standing long jump* (SW) represents strength resp. explosive power. *Sit ups* (SU) and *pull ups* (LS) represent strength endurance. *Jumping sideways* (SHH) and *balancing backward* (BAL) represent coordination under time and precision pressure. *Forward bend* (RB) represents flexibility and *a* 6-min *run* (RUN) around the volleyball court aerobic endurance. Speed was measured manually and by means of light gate measurements, which are abbreviated as SP (HS) and SP (LG). The two approaches were considered separately in the analyses. The reasons for this were that manual timing may be affected by variability in the assessor's reaction time, potentially leading to minor deviations from light-gate measurements. However, these deviations are likely small and unsystematic, although they could represent a source of data variability. The motor performance test battery is well described in respective publications (Bös, 2009, 2016). Furthermore, anthropometric data is shown in Table 1.

**Table 1:** Anthropometric data (AD, anthropometric data, M, mean value, SD, standard deviation, m, male, f, female, BMI, body-mass-index, *G*1 = 9.00–9.24 years, *G*2 = 9.25–9.49 years, *G*3 = 9.50–9.74 years, *G*4 = 9.75–9.99 years, *G*5 = 10.00–10.24 years, *G*6 = 10.25–10.49 years, *G*7 = 10.50–10.74 years, *G*8 = 10.75–10.99 years).

AD	*m/f*	9-year-olds				10-year-olds			
		*G*1	*G*2	*G*3	*G*4	*G*5	*G*6	*G*7	*G*8
		*M ±SD*	*M ±SD*	*M ±SD*	*M ±SD*	*M ±SD*	*M ±SD*	*M ±SD*	*M ±SD*
Body weight (kg)	m	32.6 ± 5.2	33 ± 5.6	33.6 ± 5.6	34.5 ± 5.9	35.2 ± 6.2	36.1 ± 7.1	37.2 ± 8.0	37.5 ± 7.4
	f	32.1 ± 6.0	32.4 ± 5.8	33.5 ± 6.4	33.9 ± 6.4	35 ± 6.7	36.3 ± 7.4	38.4 ± 8.8	39.1 ± 8.1
Body height (cm)	m	138.5 ± 5.8	138.9 ± 5.8	140.1 ± 5.9	141.4 ± 6.0	142.4 ± 6.2	143.4 ± 6.4	144.4 ± 6.4	145.1 ± 6.6
	f	137.4 ± 5.7	138.3 ± 6.0	139.7 ± 6.5	140.6 ± 6.5	142.0 ± 6.5	143.6 ± 6.8	145.4 ± 7.4	146.8 ± 7.5
BMI	m	16.9 ± 2.0	17 ± 2.2	17 ± 2.1	17.2 ± 2.2	17.3 ± 2.4	17.4 ± 2.6	17.7 ± 3.1	17.7 ± 2.8
	f	16.9 ± 2.3	16.8 ± 2.3	17 ± 2.4	17.1 ± 2.4	17.3 ± 2.6	17.5 ± 2.7	18.1 ± 3.3	18 ± 2.8

### 2.3 Procedure

The assessment was carried out by trained test supervisors or trained sports teachers, who assessed children in 45 min with standardized test materials. For a standardized warm-up, participants ran two laps around the volleyball court in a standard sports gym. The assessment began with the test item sprint, while the 6-min run was completed as the last discipline to avoid data falsification due to fatigue. The remaining test items were carried out in station mode. Subjective rating errors were minimized through standardized test procedures, as tasks do not require assessor interpretation. Data were processed and interpreted using web-based software (Bös, 2016). No changes occurred in testing protocols or GMT 6-18 norms during the assessed study period.

### 2.4 Data collection

Raw data of each individual test were recorded by the supervisors in standardized data entry forms by paper-pencil (*Z*-scores were calculated from the raw data of each movement item to normalize the test results (range 70–130). *Z*-scores were derived from age- and sex-specific normative data (MT1/GMT) (Bös, 2009). While this standardization accounts for age-related differences and may attenuate observable age effects, this was addressed by additionally analyzing raw scores alongside standardized values. Performance can subsequently be divided into five different performance classes and percentile ranks. To receive a recommendation for an NRW sports school, a minimum score of 820 points (total *Z*-score) is required. The final selection will be made based on school-specific rankings, subject to available spots.

### 2.5 Statistical analysis

Statistical analyses were carried out using IBM SPSS Statistics program versions 29.0 and 31.0 (IBM Corp, Armonk, NY, USA). After data cleansing, the entire sample was divided into eight groups (*G*1–*G*8) based on chronological age in decimal numbers (*G*1 = 9.00–9.24 years, *G*2 = 9.25–9.49 years, *G*3 = 9.50–9.74 years, *G*4 = 9.75–9.99 years, *G*5 = 10.00–10.24 years, *G*6 = 10.25–10.49 years, *G*7 = 10.50–10.74 years, *G*8 = 10.75–10.99 years). Age groups were constructed relative to individual test dates, as the test dates varied and were spread over approximately half a year. Departing from the quarterly classification based on the beginning of the year that is commonly used in RAE-research, the present study used the time of testing as the reference point, as this allows age-related differences to be captured more precisely in direct relation to the actual testing situation and the specific time of performance assessment.

Variance analyses were carried out to be able to differentiate between the correlations between the age groups and the raw scores of the test items. The degrees of freedom (df), the *F* value (*F*), the significance (*p*) and the effect size Eta-squared (η^2^) were considered to determine whether there were significant differences between the raw data of the test items and the age groups. A correlation analysis according to Spearman was applied to determine relations between calendar age and motor performance. To determine anthropometric relations to overall *Z*-scores and test items, Pearson correlations were applied. The strength of correlations was classified according to Cohen (2013) (|ρ| < 0.10 no correlation, 0.10 ≤ |ρ| < 0.30 low correlation, 0.30 ≤ |ρ| < 0.50 medium correlation, |ρ| ≥0.50 high correlation). With regard to the significance level, alpha < 0.05 was considered significant (Cohen, 2013). The interpretation of effect size Eta-squared (η^2^) was carried out according to Cohen (2013) as follows η^2^ < 0.01 no effect, 0.01 ≤ η^2^ < 0.06 small effect, 0.06 ≤ η^2^ < 0.14 medium effect, η^2^ ≥ 0.14 large effect. Due to the large sample size, effect sizes were prioritized over *p-*values to provide a more meaningful interpretation of the results. Additionally, generalized linear models (GENLIN) with normal distribution and identity link function were conducted to further examine the association between age (in decimal numbers in relation to individual test date) and BMI and test performance. Gender was included as a covariate to control for potential confounding effects. Robust (sandwich) standard errors were applied to account for heteroscedasticity. Visual inspection of Q–Q plots of standardized Pearson residuals suggested that the assumption of normality was met.

To assess the distribution of the recommendation and admission rates for NRW sports schools between the age groups, a chi-square test was performed. Hereby, degrees of freedom (df), Pearson chi-square (χ^2^), Cramer's *V* (*V*) and significance (*p*) were considered. The effect size of Cramer's *V* (*V*) is interpreted according to Cohen (2013) and referred to as follows: *V* < 0.10 no effect, 0.10 ≤ *V* < 0.30 small effect, 0.30 ≤ *V* < 0.50 medium effect, *V* ≥ 0.50 large effect. Furthermore, a binary logistic regression analysis was conducted as a sensitivity analysis to examine whether age, sex and BMI were associated with the likelihood of selection (0 = non-selected, 1 = selected). Gender was treated as a categorical variable, while BMI and age were included as continuous predictors. Model fit was assessed using the Hosmer–Lemeshow goodness-of-fit test and Nagelkerke's *R*^2^. The discriminative ability of the model was evaluated using the area under the receiver operating characteristic curve (AUC). Predicted probabilities were derived from the logistic regression model and used to construct the ROC curve. Odds ratios (ORs) with 95% confidence intervals (CIs) were reported for all predictors.

## 3 Results

For this in-depth analysis, the following study examined data from a total of 19,179 fourth-grade schoolchildren (9–10 years old) over the period from 2008 to 2022 for possible correlations between chronological age and motor performance. For a closer examination, four age groups (quarters) were created per year group, which were numbered consecutively from younger to older (*G*1–*G*8). All groups correspond to intervals of 0.249 years each. *G*1 (9.00–9.249 years) comprised the youngest, and *G*8 (10.75–10.99 years) comprised the oldest participants.

The average age of the 9-year-olds at the time of the study was 9.58 ± 0.24 years (male: *m* = 9.58, *SD* = 0.24; female: *m* = 9.57, *SD* = 0.24), and the average age of the 10-year-olds was 10.26 ± 0.24 years (male: *m* = 10.25, *SD* = 0.24; female: *m* = 10, *SD* = 0.25). With increasing age group, weight, height and BMI increased continuously from *G*1 to *G*8. Table 1 shows the anthropometric data of the included 9- to 10-year-old children.

### 3.1 Calendar age and motor performance

In relation to calendar age and motor performance, overall *Z*-scores show higher values for 9-year-olds than 10-year-olds, and girls have slightly higher mean values than boys in both age groups of the 9- and 10-year-olds. Across the groups, a slight decreasing trend in *Z*-scores is observable, particularly among the 10-year-olds from *G*5 to *G*8. Total *Z*-scores (*M* ± *SD*) differentiated by age group (9-year-olds vs. 10-year-olds), gender, and groups (*G*1–*G*8) are presented in Table 2.

**Table 2:** Overall *Z*-values in age groups (M, mean value; SD, standard deviation; m, male; f, female; *G*1 = 9.00–9.24 years, *G*2 = 9.25–9.49 years, *G*3 = 9.50–9.74 years, *G*4 = 9.75–9.99 years, *G*5 = 10.00–10.24 years, *G*6 = 10.25–10.49 years, *G*7 = 10.50–10.74 years, *G*8 = 10.75–10.99 years).

m/f	9-year-olds				10-year-olds				Total
	*G*1	*G*2	*G*3	*G*4	*G*5	*G*6	*G*7	*G*8	
	*M ±SD*	*M ±SD*	*M ±SD*	*M ±SD*	*M ±SD*	*M ±SD*	*M ±SD*	*M ±SD*	*M ±SD*
m	873 ± 42.3	874.8 ± 42.8	878.3 ± 43.2	879.7 ± 44.3	858.8 ± 45.3	853.6 ± 47.9	845.1 ± 53	848 ± 52.1	869.3 ± 46.3
	877.5 ± 51.2				855.1 ± 47.7				
f	881.6 ± 44.3	878 ± 46.5	883.1 ± 50.2	882.5 ± 47.7	860 ± 50.8	852 ± 51.5	843.5 ± 53.8	842.2 ± 50.7	871.7 ± 51.2
	882 ± 47.9				854.1 ± 51.8				

GENLIN analyses, further examining the association between age, BMI, gender and overall test performance, show BMI were negatively associated with overall test performance, indicating that higher values were linked to lower test scores. Age was also negatively associated with the overall test performance (BMI: *B* = −6.83, *SE* = 0.15, Wald χ^2^(1) = 2,053.0, *p* = < 0.001, 95% *CI* [−7.12, −6.53], age: *B* = −20.31, *SE* = 0.82, Wald χ^2^(1) = 617.30, *p* = < 0.001, 95% *CI* [−21.91, −18.71]). An differentiated analysis of the test items revealed varying effects (see Chapter 2). Gender also showed a significant effect, with boys demonstrating higher test scores than girls after controlling for age and BMI (*B* = 2.24, *SE* = 0.69, Wald χ^2^(1) = 10.47, *p* = 0.001, 95% *CI* [.88, 3.60]).

**Figure 1:**
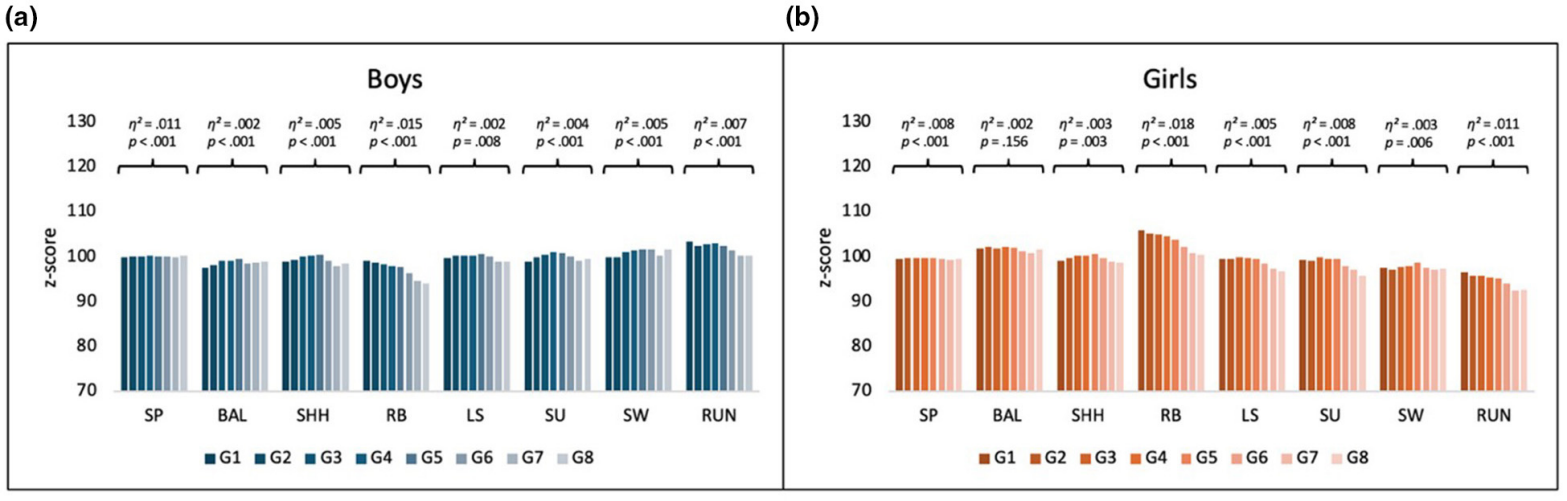
**(a, b)** Motor performance values (*Z*-scores) of boys and girls in 8 motor test items, 20 m sprint (SP), balancing backward (BAL), jumping sideways (SHH), torso bend (RB), push-ups (LS), sit-ups (SU), standing long jump (SW) and a 6-min run (RUN).

Figures 1a, b shows the distribution of the reached values (*Z*-scores) of 8 age groups in all 8 motor performance test items.

In the overall sample, both boys and girls show statistically highly significant differences (*p* < 0.01) in the majority of motor performance items. An exception is found in the balancing item for girls, where no significant difference is observed (*F* = 1.52, *p* = 0.156, η^2^ = 0.002). Small effect sizes are evident in Sprint HS (boys: *F* = 5.68, *p* < 0.001, η^2^ = 0.011; girls: *F* = 3.49, *p* < 0.001, η^2^ = 0.012), trunk flexibility (boys: *F* = 27.58, *p* < 0.001, η^2^ = 0.015; girls: *F* = 17.20, *p* < 0.001, η^2^ = 0.018), and in the 6-min run for girls (*F* = 11.01, *p* < 0.001, η^2^ = 0.011). *Post hoc* analyses provide further insight into group-specific differences. For Sprint (HS), boys in groups *G*1–*G*3 (aged 9.00–9.74 years) performed significantly lower than those in group *G*5 (10.00–10.24 years; *p* < 0.006), indicating superior performance in the older cohort. Among girls, significant differences were found between *G*2 (9.25–9.49 years) and the older groups *G*5 and *G*6 (10.00–10.49 years; *p* < 0.003), again in favor of the older participants. A consistent pattern is also observed in trunk flexibility: both boys and girls in groups *G*1–*G*4 (aged 9.00–9.99 years) achieve significantly better results than the older in groups *G*5–*G*8 (aged 10.00–10.99 years; *p* < 0.05), suggesting a decline in flexibility performance with increasing age. Similarly, in the 6-min run, the younger girls (*G*1–*G*4) outperform their older peers in groups *G*6–*G*8 (*p* < 0.05), further highlighting age-related performance differences in the motor performance items.

A separate analysis of the age groups of 9- and 10-year-old children reveals distinct patterns regarding statistical significance and effect sizes in the motor performance tests conducted. Among the 9-year-old boys, almost all test items show statistically significant differences (*p* < 0.05), with the exception of *push-ups* (*p* = 0.336). However, despite these statistical significances, none of the items exhibit meaningful effect sizes, indicating limited practical relevance of the observed differences. In contrast, for 9-year-old girls, statistically significant differences are only evident for *sprint* (*p* = 0.040) and *lateral jumping* (*p* = 0.041). For the 10-year-old boys, statistically significant differences are observed in nearly all items (*p* < 0.05), except for *balancing* (*p* = 0.073) and *standing long jump* (*p* = 0.066). A small yet notable effect size in favor of the younger boys is detected in *trunk flexibility* (*F* = 28.012, *p* < 0.001, η^2^ = 0.018). For the 10-year-old girls, all test items—except for *balancing* (*p* = 0.097)—show statistically significant differences (*p* < 0.05). Small effect sizes in favor of the younger girls are evident in *Sprint* (*F* = 7.716, *p* < 0.001, η^2^ = 0.014), *trunk flexibility* (*F* = 11.296, *p* < 0.001, η^2^ = 0.014), *sit-ups* (*F* = 11.317, *p* < 0.001, η^2^ = 0.014), and the 6-min *run* (*F* = 9.848, *p* < 0.001, η^2^ = 0.012). The raw values of all 8 motor performance items are presented in Table 3.

**Table 3:** Means and standard deviations of raw test-scores for each item [*M* = Mean value, *SD* = Standard deviation, m = male, f = female, n = subsample, 20 m sprint (SP) (HS and LG), Balancing backward (BAL), Jumping sideways (SHH), Torso bend (RB), Push-ups (LS), Sit-ups (SU), standing long jump (SW) and a 6-min run (RUN), (*G*1 = 9.00–9.24 years, *G*2 = 9.25–9.49 years, *G*3 = 9.50–9.74 years, *G*4 = 9.75–9.99 years, *G*5 = 10.00–10.24 years, *G*6 = 10.25–10.49 years, *G*7 = 10.50–10.74 years, *G*8 = 10.75–10.99 years)].

Test	m/f	*n*	9-year old				10-year old			
			*G*1	*G*2	*G*3	*G*4	*G*5	*G*6	*G*7	*G*8
			*M ±SD*	*M ±SD*	*M ±SD*	*M ±SD*	*M ±SD*	*M ±SD*	*M ±SD*	*M ±SD*
SP HS (Sec.)	m	3,581	4.06 ± 0.22	4.05 ± 0.25	4.02 ± 0.27	4.01 ± 0.26	4.0 ± 0.26	3.97 ± 0.28	4.04 ± 0.32	3.97 ± 0.3
	f	1,998	4.2 ± 0.28	4.23 ± 0.27	4.16 ± 0.28	4.16 ± 0.29	4.13 ± 0.31	4.13 ± 0.3	4.23 ± 0.27	4.17 ± 0.39
SP LG (Sec.)	m	8,939	3.73 ± 0.21	3.72 ± 0.21	3.69 ± 0.22	3.68 ± 0.22	3.69 ± 0.23	3.71 ± 0.25	3.73 ± 0.29	3.68 ± 0.28
	f	4,634	3.83 ± 0.22	3.82 ± 0.22	3.81 ± 0.25	3.79 ± 0.24	3.79 ± 0.26	3.84 ± 0.28	3.88 ± 0.3	3.85 ± 0.25
BAL (steps)	m	12,538	36.6 ± 8.8	37.1 ± 8.1	37.8 ± 7.9	37.9 ± 8.1	38.1 ± 8.1	37.4 ± 8.3	37.6 ± 8.1	37.7 ± 8.8
	f	6,641	40.1 ± 7.4	40.3 ± 7.4	40.1 ± 7.7	40.4 ± 7.4	40.2 ± 7.5	39.6 ± 7.6	39.2 ± 7.7	39.9 ± 7.2
SHH (rep.)	m	12,538	34.9 ± 6.8	35.2 ± 6.8	35.7 ± 6.9	35.9 ± 6.9	36 ± 6.9	35.1 ± 6.9	34.3 ± 6.9	34.7 ± 7.2
	f	6,641	35.1 ± 6.5	35.4 ± 6.7	35.9 ± 6.7	35.8 ± 6.5	36.1 ± 6.6	35.5 ± 6.2	34.9 ± 6.7	34.8 ± 6.6
RB (cm)	m	12,537	2.3 ± 5.8	2.0 ± 6.0	1.7 ± 6.0	1.4 ± 6.2	1.3 ± 6.3	0.4 ± 6.4	−0.8 ± 7.1	−1.2 ± 6.8
	f	6,641	6.9 ± 6.3	6.3 ± 6.6	6.2 ± 6.6	5.9 ± 6.6	5.4 ± 6.7	4.4 ± 7.2	3.5 ± 7.5	3.2 ± 7.4
LS (rep.)	m	12,538	14 ± 4.1	14.3 ± 4.3	14.3 ± 4.4	14.3 ± 4.3	14.4 ± 4.4	14.2 ± 4.3	13.7 ± 4.6	13.7 ± 4.9
	f	6,641	13.9 ± 4.3	13.9 ± 4.2	14.1 ± 4.5	14 ± 4.3	13.9 ± 4.5	13.5 ± 4.4	13 ± 4.7	12.7 ± 4.7
SU (rep.)	m	12,538	22 ± 4.6	22.5 ± 4.9	22.8 ± 4.8	23.1 ± 4.9	23.0 ± 5.0	22.6 ± 5.1	22.1 ± 5.5	22.3 ± 5.4
	f	6,641	22.2 ± 5.2	22.1 ± 5.1	22.5 ± 5.3	22.3 ± 5.0	22.3 ± 5.3	21.5 ± 5.4	21.1 ± 5.2	20.4 ± 5.5
SW (cm)	m	12,538	149.8 ± 16.6	150.0 ± 17.1	151.9 ± 17.7	152.8 ± 17.8	153.1 ± 18.5	153.3 ± 19.6	150.7 ± 21.5	153.0 ± 21.3
	f	6,641	145.6 ± 16.6	144.7 ± 18.1	145.7 ± 19.2	146.3 ± 18.3	147.8 ± 19.6	145.5 ± 21.1	144.6 ± 22.2	145.2 ± 21.7
6-min –run (m)	m	12,537	1168.1 ± 101.5	1,154.8 ± 111.2	1,160.4 ± 109.1	1,163.1 ± 111.7	1,154.2 ± 116.9	1,143.3 ± 122.3	1,128.3 ± 136.5	1,129.1 ± 138.1
	f	6,641	1,084 ± 100.1	1,074.9 ± 107.9	1,074.7 ± 117.1	1,069.8 ± 109.8	1,067.3 ± 118.6	1,051.4 ± 121.9	1,032 ± 144.3	1,035.7 ± 122.5

GENLIN analyses, further examining the association between age, BMI, gender and raw test values, showed associations between age and raw test values in favor of the older children for BAL (*B* = 0.71, *SE* = 0.14, Wald χ^2^(1) = 25.50, 95% *CI* [0.43, 0.98], *p* = < 0.001), SHH (*B* = 0.28, *SE* = 0.12, Wald χ^2^(1) = 5.20, 95% *CI* [0.04, 0.52], *p* = 0.023) and SW (*B* = 2.94, *SE* = 0.32, Wald χ^2^(1) = 81.94, 95% *CI* [2.30, 3.57], *p* = < 0.001). All further associations were negative, therefore in favor of the younger children of the test sample, or, for *push-ups* and *sit-ups*, effects were statistically insignificant (LS: *p* = 0.294, SU: *p* = 0.712), underlining the previously reported heterogeneity in test performance with respect to calendar age (the full table can be found in Appendix A1).

### 3.2 Relation between anthropometric properties and performance in individual tests and overall *Z*-score

Table 4 shows Pearson correlation coefficients (*r*) between body weight (kg) and the performance in the individual test items as well as the overall *Z*-score, separately for girls and boys. For both girls and boys, significant correlations were found between body weight and performance in all motor test items (*p* < 0.001). Positive correlations were found for sprint performance, whereas predominantly negative correlations emerged for the remaining motor performance items and for the overall *Z*-score. The strongest associations were observed for the 6-min run and the overall *Z*-score in both girls and boys. High levels of statistical significance are partly attributable to the large sample size, resulting in even small to moderate correlation coefficients reaching statistical significance.

**Table 4:** Correlations between body weight (kg) and performance in individual test items and overall *Z*-score (m = male, f = female).

m/f	SP		BAL		SHH		RB		LS		SU		SW		6-min		Overall Z-score	
	*r*	*p*	*r*	*p*	*r*	*p*	*r*	*p*	*r*	*p*	*r*	*p*	*r*	*p*	*r*	*p*	*r*	*p*
m	0.202	< 0.001	−0.239	< 0.001	−0.151	< 0.001	−0.081	< 0.001	−0.186	< 0.001	−0.121	< 0.001	−0.198	< 0.001	−0.368	< 0.001	−0.333	< 0.001
f	0.188	< 0.001	−0.287	< 0.001	−0.168	< 0.001	−0.142	< 0.001	−0.199	< 0.001	−0.207	< 0.001	−0.204	< 0.001	−0.368	< 0.001	−0.368	< 0.001

Correlations between body height (cm) and performance in individual test items and overall *Z*-Score show, for both girls and boys, mostly small but statistically significant correlations between body height (cm) and performance across all motor test items (see Table 5). Predominantly negative associations were found for balance, mobility and strength-related test items, whereas small positive correlations were observed for *standing long jump* (SW) in both girls and boys. Correlations with the overall *Z*-score were weakly negative for both girls and boys. Similar to body weight calculations, the high level of statistical significance is partly attributable to the large sample size.

**Table 5:** Correlations between body height (cm) and performance in individual test items and overall *Z*-score (m = male, f = female).

m/f	SP	BAL		SHH		RB		LS		SU		SW		6-min		Overall Z-score		
	*r*	*p*	*r*	*p*	*r*	*p*	*r*	*p*	*r*	*p*	*r*	*p*	*r*	*p*	*r*	*p*	*r*	*p*
m	−0.042	< 0.001	−0.107	< 0.001	−0.056	< 0.001	−0.135	< 0.001	−0.118	< 0.001	−0.022	0.015	0.109	< 0.001	−0.082	< 0.001	−0.130	< 0.001
f	−0.085	< 0.001	−0.129	< 0.001	−0.036	0.003	−0.156	< 0.001	−0.132	< 0.001	−0.081	< 0.001	0.102	< 0.001	−0.095	< 0.001	−0.149	< 0.001

The GENLIN analyses of raw test values revealed a positive association between higher BMI and increased *sprint* time in both HS and LG measurements after controlling for gender and age (HS: *B* = 0.03, *SE* = 0.00, Wald χ^2^(1) = 339.33, 95% *CI*. [0.03, 0.04], *p* = < 0.001, LG: *B* = 0.03, SE = 0.00, Wald χ^2^(1) = 760.64, 95% *CI*. [0.03, 0.03], *p* = < 0.001). Higher values in sprint performance indicate less motor performance in speed velocity.

### 3.3 Relation between age group (G1–G8) and recommendation and admission to an NRW sports school

Table 6 shows the results of a chi-square test to analyze the relation between the recommendation and admission to a sports school and age groups (*G*1–*G*8). For the total sample of boys, a small but statistically highly significant correlation was found between the age groups and the recommendation for a sports school in favor of the younger children (*G*1–*G*4, aged 9.00–9.99 years) compared to their older peers (χ^2^ = 427.65, *V* = 0.185, *p* < 0.001). This correlation was also evident among female participants in favor of the younger girls born in *G*1–*G*4 (χ^2^ = 289.29, *V* = 0.209, *p* < 0.001).

**Table 6:** Chi-square test examining the association between the recommendation and admission to an NRW sports school and the age group (*G*1–*G*8), *df* = degrees of freedom, χ^2^ = Pearson-Chi-square, *V* = Cramers V, *p* = significance, m = male, f = female, ^*^ = *p* < 0.05.

Group	m/f	df	Recommendation			Admission		
			χ^2^	*V*	*p*	χ^2^	*V*	*p*
Total	m	7	427.65	0.185	< 0.001^*^	31.03	0.059	< 0.001^*^
	f	7	289.29	0.209	< 0.001^*^	27.15	0.076	< 0.001^*^
9-year-olds	m	3	6.66	0.029	0.083	27.42	0.069	< 0.001^*^
	f	3	0.49	0.011	0.920	14.17	0.067	0.003^*^
10-year-olds	m	3	40.08	0.094	< 0.001^*^	3.55	0.033	0.314
	f	3	53.81	0.148	< 0.001^*^	8.39	0.072	0.039^*^

Within the age group of 9-year-olds, no significant correlations could be found between age groups and recommendation to a sports school for either boys or girls (m: χ^2^ = 6.66, *V* = 0.029, *p* = 0.083; f: χ^2^ = 0.49, *V* = 0.011, *p* = 0.920). In the age group of 10-year-old boys, the differences are highly significant but do not account for any effect (χ^2^ = 40.08, *V* = 0.094, *p* < 0.001). The results for 10-year-old girls are also highly significant (χ^2^ = 53.81, *V* = 0.148, *p* < 0.001). The effect size (*V* = 0.148) shows a small effect size in favor of the older ones. The results also show a highly significant correlation between age group and admission to an NRW sports school in the overall sample for both boys (χ^2^ = 31.03, *V* = 0.059, *p* < 0.001) and girls (χ^2^ = 27.15, *V* = 0.076, *p* < 0.001). However, the effect sizes (*V* < 0.10) indicate a negligible practical effect. Among 9-year-olds, the correlation is highly significant for boys (χ^2^ = 27.42, *V* = 0.069, *p* < 0.001) and significant for girls (χ^2^ = 14.17, *V* = 0.067, *p* = 0.003), with low effect sizes. There are differences among the 10-year-olds: While there is no significant correlation for boys (χ^2^ = 3.55, *V* = 0.033, *p* = 0.314), it is significant for girls (χ^2^ = 8.39, *V* = 0.072, *p* = 0.039) but also without a practical relevant effect.

Furthermore, a binary logistic regression assessed the robustness of these findings. The overall model was statistically significant, χ^2^(3) = 1,859.49, *p* = < 0.001, explaining a modest proportion of variance (Nagelkerke *R*^2^ = 0.17). Age (in decimal numbers in relation to individual test date) was negatively associated with selection (*B* = −1.0, *SE* = 0.06, *OR* = 0.37, 95% *CI* [0.332 −0.411], *p* = < 0.001). BMI also showed a negative association (*B* = −0.30, *SE* = 0.01, *OR* = 0.74, 95% *CI* [0.727 −0.752], *p* = < 0.001). Gender was a positively significant, but a minor evident predictor in favor of boys (*B* = 0.12, *SE* = 0.05, *OR* = 1.13, *CI* [1.028–1.235], *p* = < 0.001), indicating that boys had slightly higher odds of being selected than girls. Ties in predicted probabilities were present, the model nevertheless showed acceptable discrimination (*AUC* = 0.72). Although the Hosmer–Lemeshow test was significant (*p* = < 0.001), this was interpreted with caution given the sensitivity of the test to sample size. The percentage distribution of recommendations and admissions to NRW sports schools of the examined children is presented in Table 7.

**Table 7:** Recommendation and admissions to NRW sports schools (m = male, f = female, o = overall, *G*1 = 9.00–9.24 years, *G*2 = 9.25–9.49 years, *G*3 = 9.50–9.74 years, *G*4 = 9.75–9.99 years, *G*5 = 10.00–10.24 years, *G*6 = 10.25–10.49 years, *G*7 = 10.50–10.74 years, *G*8 = 10.75–10.99 years).

NRW Sports school		m/f/o	9-year-olds				10-year-olds			
			*G*1	*G*2	*G*3	*G*4	*G*5	*G*6	*G*7	*G*8
Recommendation	Number of recommendations	m	513 (4.1%)	1,772 (14.1%)	2,367 (18.8%)	2,583 (20.6%)	2,020 (16.1%)	975 (7.8%)	364 (2.9%)	234 (1.9%)
		f	339 (5.1%)	924 (13.9%)	1,213 (18.3%)	1,296 (19.5%)	1,026 (15.4%)	489 (7.4%)	213 (3.2%)	141 (2.1%)
		o	11,007 (57.4%)				5,462 (28.5%)			
	% within the age group	m	89.1%	89.8%	91.3%	91.4%	82%	75.8%	72.7%	72.9%
		f	89.9%	89.3%	90.1%	89.9%	81.6%	75.7%	65.3%	66.2%
		o	90.5%				77.9%			
Admission	Number of admissions	m	208 (2.3%)	774 (8.6%)	1,068 (11.9%)	1,258 (14.0%)	967 (10.8%)	551 (6.1%)	182 (2.0%)	96 (1.1%)
		f	154 (3.2%)	470 (9.9%)	659 (13.9%)	677 (14.2%)	549 (11.5%)	256 (5.4%)	95 (1.9%)	59 (1.2%)
		o	5,268 (38.4%)				2,755 (20.1%)			
	% within the age group	m	50.6%	53.8%	56.4%	61.1%	56%	58.4%	58.3%	51.9%
		f	53.8%	60.4%	64.9%	64.1%	62.2%	54.4%	56.9%	61.5%
		o	58.9%				57.6%			

## 4 Discussion

In this investigation, the overall *Z*-score and the raw data of the 8 individual test items of GMT 6–18 were analyzed with regard to a possible effect of calendar age plus recommendation and admission to an NRW sports school including the examination of effects of anthropometric properties. The results show significant differences in motor performance test items due to the large sample size; therefore, effect sizes and multivariate analyses were considered more relevant (Kornmann et al., 2024). In summary, older children seem to have a small advantage in terms of speed (20-m-sprint), due to the fact that strength is the performance-determining factor in this test item. This result seems reasonable against the background of the anthropometric data, which show a continuous increase of height, weight and BMI from younger to older cohorts and the observation, that speed of movement develops particularly quickly up to the age of 10, but this could not be confirmed by multivariate analyses (Ahnert, 2005). However, positive correlations between *Sprint* performance and body weight as well as *Standing long jump* and body height are in line with this interpretation.

Likewise, both older boys and girls have a disadvantage in terms of trunk flexibility, presumably due to a decline in flexibility with increasing age, which is consistent with the results of other studies where this test item is used to assess mobility (Drenowatz et al., 2021; Starker et al., 2007). However, this contrasts with the general assumption that mobility increases until puberty (Baur, 1994). In terms of aerobic endurance, younger girls (9-year-olds) outperformed older girls (10-year-olds). For other strength and coordination-specific performance items, no practical effect was found in favor of older or younger cohorts. In regard to further anthropometric analyses, negative associations were found between body weight and height in relation to balance, mobility and strength-related test items. Accordingly, the present data support the hypotheses formulated in the introduction despite some contrasting findings for individual test items. However, these are rather divergent, so that advantages associated with RAEs cannot be assumed. With respect to the limitations of the study, an inherent selection bias should be considered in this sample, as the included boys and girls were assessed within a competitive admission process to enroll at an NRW sports school, potentially yielding a subgroup that is more motivated and higher-performing than the general population, which may limit the generalizability of the findings.

In conclusion, based on the effect sizes of the raw value analysis of the 8 performance test items, no systematic effect in advantage for older children could be verified. Within the four cohorts of each 9-year-old and 10-year-old children, a small effect size in favor of the older girls was observed in the sprint (HS) performance test of the 9-year-old girls. Among the 10-year-old boys, a small effect was detected in trunk flexibility in favor of the younger boys, and for the 10-year-old girls, small effects were found in sprint, sit-ups, trunk flexibility and the 6-min run, the first two in favor of the older ones, and the second two in favor of the younger ones. The raw data analysis of GMT 6–18 showed robustness in its diagnostic value and susceptibility to RAEs, combining the results of the individual performance test items. Examining the association between the recommendation to an NRW sports school and age groups, the results indicate no relevant effect in age and an advantage of recommendation for and admission to an NRW sports school. Logistic regression indicated that boys had slightly higher odds of being selected compared to girls.

Furthermore, the results proved that it would be advisable to take a more in-depth approach to the raw values in addition to analyzing the *Z*-scores and incorporating raw scores into selection decisions. Clearer differences in favor of younger or older cohorts emerged in the raw value analyses, which could not have been captured in this precise order with a regular *Z*-score analysis solely.

Implications for policy and practice may include accounting for the susceptibility of specific test items to anthropometric bias and avoiding reliance on aggregate scores alone. Furthermore, effective talent development may benefit from a precise and differentiated diagnostic basis to guide targeted training and monitor progression. Although observed RAEs were small and unsystematic, they might still be practically relevant and should be taken into consideration, as subtle biases could accumulate in selective decision-making contexts. Schools might adjust recruitment processes to further reduce bias by complementing the 820 composite score with measures that ensure a balanced selection across all four birth quartiles or by accounting for individuals' proximity to the growth spurt and balanced gender distribution. Additionally, incorporating training years within a structured, professional environment into decision-making processes or analytical models may further help to mitigate potential bias.

As children enter puberty, it would be useful to critically examine both the data collection items and to identify the potential to mathematically address the problem. Research in the field of soccer showed that the RAEs became more pronounced with the onset of puberty (Bozděch et al., 2023). The research indicates that there is a medium risk of RAEs occurring in relatively older youth soccer players, which decreases once they reach the adult age group (Bozděch et al., 2023). The data examined support these findings in that 9- and 10-year-old children showed a balanced motor performance profile in the overall test results. In a study focusing on RAEs in French alpine skiing, RAEs were addressed by examining the distribution of birth trimesters and competition performances in adolescent competitors over a long-term period (De Larochelambert et al., 2022). A linear relationship between the distribution of performances and months provided a calibration coefficient that allows performances to be reweighted considering the effect of possible RAEs. Furthermore, the determination of age at peak height velocity (APHV) could complement examinations to account for developmental fluctuations in physical performance (Müller et al., 2015). Mirwald's prediction equations for determining APHV include leg length and sitting height to account for the different proportions between the trunk and extremities (Mirwald et al., 2002). It does not require a significant amount of time for motor performance testing and seems acceptable for additional consideration, but limitations of the equations with early and late maturing boys and girls must also be taken into consideration (Kozieł and Malina, 2018). Moreover, it seems necessary to conduct further analyses to examine the longitudinal development of children selected for sports schools on the basis of the generic motor performance test in terms of their later sporting success. It would be particularly interesting to see whether RAE is evident in successful athletes compared to less successful ones in this sample. Furthermore, the data could contribute to the predictive value of the GMT for future performance (see e.g., Hohmann and Siener, 2021).

## 5 Conclusions

The aim of this study was to investigate whether talent selection based on a generic motor performance test (GMT 6–18) in 9- and 10-year-old children was affected by bias related to RAEs. The findings of the present study indicate that the selection process of NRW sports schools shows minimum systematic bias by children's chronological age. The findings suggest that GMT 6–18 can be considered a rather fair method for early talent identification and may be applied confidently in practical settings without concern for age-related disadvantage. Although the RAEs observed in this cohort were comparatively small, their practical significance should not be disregarded, as even subtle biases may translate into meaningful disparities within high-stakes selection contexts. Additionally, robust performing approaches require further improvement to enhance accuracy, indicating that current assessment approaches and statistical models still require further refinement to ensure fair decision-making. Further research is needed to verify the findings of this study, including a closer examination of both biological and training age. Furthermore, talent diagnostics considering anthropometric aspects for a specification of strength and power per unit of body mass or the usage of complementary mathematical solutions to reweight the distribution of performances and birth months could be viable to address RAEs in competitive youth sports.

## Data Availability

The data analyzed in this study is subject to the following licenses/restrictions: They can be provided on reasonable request. Requests to access these datasets should be directed to i.stolz@dshs-koeln.de.
