# Supplementary material for: The role of relative age effects in generic motor skill diagnostics using the German motor test (6–18): diagnostic outcomes and recruitment of 9–10-year-old children to sports schools in Germany
Source: Front Psychol. 2026 May 8;17:1814502. doi: 10.3389/fpsyg.2026.1814502 (PMC13195887; doi:10.3389/fpsyg.2026.1814502)
Supplement: Supplementary file 1 [file Supplementary_file_1.docx]

**The Role of Relative Age Effects (RAEs) in Generic Motor Skill Diagnostics Using the German Motor Test (6–18): Diagnostic Outcomes and Recruitment of 9–10-Year-Old Children to Sports Schools in Germany**

# Stolz Isabel^1,*^, Bokeloh Tabea^1+^, Feldhaus Chiara^2,+^, and Bös Klaus^2,+^

^1^Institute of Movement and Neurosciences, German Sport University Cologne, 50933 Cologne, Germany

^2^Research Center for Physical Education and Sport for Children and Adolescents, Karlsruhe Institute of Technology, 76131 Karlsruhe, Germany

**Correspondence:**

Corresponding author

^*^i.stolz@dshs-koeln.de

^+^these authors contributed equally to this work

**Appendix 1**

| **Predictor** | **B** | **SE** | **95% CI** | **Wald χ²** | ***p*** |
| --- | --- | --- | --- | --- | --- |
| **SP HS (Sec.)** | | | | | |
| Gender | .15 | .01 | .14 - .17 | 382.24 | <.001 |
| BMI | .03 | .00 | .03 - .04 | 339.33 | <.001 |
| Age | -.08 | .01 | -.10 - -.06 | 74.29 | <.001 |
| **SP LG (Sec.)** | | | | | |
| Gender | .11 | .00 | .11 - .12 | 721.31 | <.001 |
| BMI | .03 | .00 | .03 - .03 | 760.64 | <.001 |
| Age | -.02 | .01 | -.03 - -.01 | 13.82 | <.001 |
| **BAL (Steps)** | | | | | |
| Gender | 2.39 | .11 | 2.17 – 2.62 | 438.42 | <.001 |
| BMI | -.86 | .03 | -.91 - -.81 | 1048.79 | <.001 |
| Age | .71 | .14 | .43 - .98 | 25.50 | <.001 |
| **SHH (rep.)** | | | | | |
| Gender | .10 | .10 | -1.00 - .300 | .94 | .332 |
| BMI | -.49 | .02 | -.526 - -.445 | 546.30 | <.001 |
| Age | .28 | .12 | .04 - .52 | 5.20 | .023 |
| **RB**  **(cm)** | | | | | |
| Gender | 4.32 | .10 | 4.12 – 4.52 | 1842.85 | <.001 |
| BMI | -.08 | .02 | -.12 - -.04 | 17.71 | <.001 |
| Age | -1.82 | .12 | -2.06 - -1.59 | 232.28 | <.001 |
| **LS**  **(rep.)** | | | | | |
| Gender | -.41 | .07 | -.55 - -.28 | 38.32 | <.001 |
| BMI | -.30 | .01 | -.33 - -.28 | 479.42 | <.001 |
| Age | -.08 | .08 | -.24 - .07 | 1.10 | .294 |
| **SU**  **(rep.)** | | | | | |
| Gender | -.65 | .08 | -.80 - -.50 | 70.22 | <.001 |
| BMI | -.36 | .02 | -.39 - -.32 | 426.08 | <.001 |
| Age | .03 | .09 | -.15 – 2.1 | .14 | .712 |
| **SLJ**  **(cm)** | | | | | |
| Gender | -6.2 | .27 | -6.75 - -5.68 | 519.12 | <.001 |
| BMI | -2.55 | .06 | -2.66 - -2.44 | 2094.58 | <.001 |
| Age | 2.94 | .32 | 2.30 - 3.57 | 81.94 | <.001 |
| **6-Minutes-run**  **(m)** | | | | | |
| Gender | -89.27 | 1.61 | -92.42 - -86.12 | 3079.25 | <.001 |
| BMI | -20.01 | .35 | -20.71 - -19.32 | 3184.72 | <.001 |
| Age | -9.91 | 1.96 | -13.75 - -6.08 | 25.64 | <.001 |

**Table A1**. Results of generalized linear models (GENLIN) with robust (sandwich) standard errors examining the association between age, BMI, and sex and raw test values.
